# Mortality Prediction in the Oldest Old with Five Different Equations to Estimate Glomerular Filtration Rate: The Health and Anemia Population-based Study

**DOI:** 10.1371/journal.pone.0136039

**Published:** 2015-08-28

**Authors:** Sara Mandelli, Emma Riva, Mauro Tettamanti, Paolo Detoma, Adriano Giacomin, Ugo Lucca

**Affiliations:** 1 Laboratory of Geriatric Neuropsychiatry, IRCCS—Istituto di Ricerche Farmacologiche “Mario Negri”, Milan, Italy; 2 Laboratory of Analysis, Ospedale degli Infermi, Biella, Italy; 3 County Cancer Registry, Local Health Authority, Biella, Italy; University Jean MONNET of SAINT-ETIENNE, UNITED STATES

## Abstract

**Background:**

Kidney function declines considerably with age, but little is known about its clinical significance in the oldest-old.

**Objectives:**

To study the association between reduced glomerular filtration rate (GFR) estimated according to five equations with mortality in the oldest-old.

**Design:**

Prospective population-based study.

**Setting:**

Municipality of Biella, Piedmont, Italy.

**Participants:**

700 subjects aged 85 and older participating in the “Health and Anemia” Study in 2007–2008.

**Measurements:**

GFR was estimated using five creatinine-based equations: the Cockcroft-Gault (C-G), Modification of Diet in Renal Disease (MDRD), MAYO Clinic, Chronic Kidney Disease Epidemiology Collaboration (CKD-EPI) and Berlin Initiative Study-1 (BIS-1). Survival analysis was used to study mortality in subjects with reduced eGFR (<60 mL/min/1.73m^2^) compared to subjects with eGFR ≥60 mL/min/1.73m^2^.

**Results:**

Prevalence of reduced GFR was 90.7% with the C-G, 48.1% with MDRD, 23.3% with MAYO, 53.6% with CKD-EPI and 84.4% with BIS-1. After adjustment for confounders, two-year mortality was significantly increased in subjects with reduced eGFR using BIS-1 and C-G equations (adjusted HRs: 2.88 and 3.30, respectively). Five-year mortality was significantly increased in subjects with eGFR <60 mL/min/1.73m^2^ using MAYO, CKD-EPI and, in a graduated fashion in reduced eGFR categories, MDRD. After 5 years, oldest old with an eGFR <30 mL/min/1.73m^2^ showed a significantly higher risk of death whichever equation was used (adjusted HRs between 2.04 and 2.70).

**Conclusion:**

In the oldest old, prevalence of reduced eGFR varies noticeably depending on the equation used. In this population, risk of mortality was significantly higher for reduced GFR estimated with the BIS-1 and C-G equations over the short term. Though after five years the MDRD appeared on the whole a more consistent predictor, differences in mortality prediction among equations over the long term were less apparent. Noteworthy, subjects with a severely reduced GFR were consistently at higher risk of death regardless of the equation used to estimate GFR.

## Introduction

Chronic kidney disease (CKD) is defined as abnormalities of kidney structure or function, present for > 3 months, with implications for health [1]. Glomerular filtration rate (GFR) is an important indicator of kidney function, however its direct measurement in routine clinical practice is often too complex and expensive [2] to assess kidney function in large, representative populations [3]. Several creatinine-based equations have therefore been developed over the last four decades in the attempt to estimate the GFR. In 1976, Cockcroft and Gault (C-G) developed a formula to estimate GFR, using data from 249 male patients aged 19–92 years, with creatinine clearance between 30 and 130 mL/min [4]. Subsequently, Rostoker et al. (2007) proposed a modified C-G formula taking into account the body surface area (BSA) to improve the accuracy of GFR estimation [5]. The Modification of Diet in Renal Disease (MDRD) formula was developed in 1999 based on a sample of 1,628 patients with CKD and a mean age of 51±13 years [6]. In 2007 this equation was re-expressed using standardized creatinine values [7]. In 2002 a new formula called the MAYO Clinic quadratic equation was developed, based on a combined sample of 580 consecutive healthy persons with a mean age of 41±11 years, and 320 consecutive patients with CKD and a mean age of 53±15 years [8]. The Chronic Kidney Disease Epidemiology Collaboration (CKD-EPI) formula was developed in 2009 on a sample of 5,504 subjects with and without CKD, with a wide age range (18–97 years), but only 78 subjects aged 80 years and older were present among the 12,150 included overall in the development group and internal and external validation samples [9].

Observing that available equations were poorly validated in elderly persons, in 2012 Schaeffner et al. developed two new equations in a community-based sample of 610 subjects aged 70 years or older (mean age 78.5 years; subjects aged 85-plus: 104). The first equation (Berlin Initiative Study 1, BIS-1) included serum creatinine, age and sex, while the second (BIS-2) also included cystatin C [10].

CKD is often diagnosed and staged according to the Kidney Disease Improving Global Outcome (KDIGO) Clinical Practice Guideline. According to KDIGO guideline, a threshold of GFR <60 ml/min/1.73 m^2^ for > 3 months indicates CKD [1]. However, it is still being debated whether this fixed eGFR threshold of <60 mL/min/1.73m^2^ should be used to identify CKD in the elderly general population [11–13].

Prevalence of decreased renal function expressed as GFR increases considerably with age[14], but little is known about its clinical significance in the oldest old, the fastest growing segment of the elderly population. In this age group, the validity of the equations estimating GFR and the association between reduced GFR and increased risk of mortality are still open issues [15,16]. To the best of our knowledge, only one study has examined the ability of diverse equations in predicting mortality in the oldest old [17] and, in general, only a few community-based studies have assessed the association of eGFR with mortality in 80 years and older subjects [18–23]. The aim of the present study was to investigate the association of all-cause mortality with reduced GFR estimated with five commonly used equations in a population-based study of subjects 85 years and older.

## Methods

### Study Population

All residents in the municipality of Biella, Piedmont, Italy, aged 85 years and older (N = 1,533) were eligible for the “Health and Anemia” study. Case ascertainment was made between May 2007 and July 2008. Details of the study have been reported elsewhere [24]. Briefly, a registered nurse visited each subject involved in the study at her/his place of residence whether home or institution to collect a blood sample for hematological and biochemical measurements together with information about demographic and clinical characteristics. One hundred and fifty nine subjects could not be traced and 183 died before blood sample. Of the 1,191 oldest old traced, 470 refused to or could not donate a blood sample and 721 agreed to participate in the “Health and Anemia” study. Serum creatinine was not available for 17 subjects and 4 subjects were excluded because they had their blood samples taken before the prevalence day, leaving 700 subjects available for analyses. Forty-one subjects had missing values for height or weight, so GFR estimated with the C-G was possible only for 659 subjects. Subjects who refused and subjects who agreed to participate in the study did not differ for age (mean age ± standard deviation [SD], respectively 89.2±3.7 and 89.4±3.7, *P* = 0.318) and there was a slightly lower percentage of women among participants (73.1% vs 78.1%, *P* = 0.052).

Deaths were ascertained using data from the Registry Office and the Local Health Authority of Biella. No reliable information was available on the causes of death.

### Measurements

A questionnaire was administered by a registered nurse in order to ascertain habits, present and past diseases, previous hospital admissions and drug therapies. The information about past and present diseases was self-reported by subjects during the interview. During the same visit, the nurse also gathered information about medication intake. Classification of diseases was based on the International Classification of Disease, Tenth Revision (ICD 10). Serum creatinine was measured using the kinetic colorimetric assay based on the Jaffe reaction, which has a higher sensitivity and better precision than the original Jaffe method. The method has been standardized against isotope dilution mass spectrometry (ID-MS) (CREA Creatinine Jaffe method, Cobas Roche Hitachi). Coefficients of variation (CV) were 2.3%, 1.5% and 1.7% at serum creatinine concentrations of 1.09 mg/dL, 1.92 mg/dL, and 3.70 mg/dL respectively.

Based on serum creatinine concentrations, GFR was estimated using the following five equations:

a) C-G [5]:

$$(140-age(years))\times weight(kg)÷(72\times creatinine(mg/dL))\times \left(\frac{1.73}{BSA}\right)\times 0.85\;if\;female$$

body surface area was calculated using DuBois formula:

$$0.007184\times {weight\;(kg)}^{0.425}\times {height\;(cm)}^{0.725}$$

b) MDRD [7]:

$$175\times {creatinine(mg/dL)}^{-1.154}\times {age(years)}^{-0.203}\times 0.742\;if\;female$$

c) MAYO [8]:

$$e^{1.911+\frac{5.249}{creatinine(mg/dL)}-\frac{2.114}{{creatinine(mg/dL)}^{2}}-0.00686\times age(years)-0.205\;if\;female}$$

d) CKD-EPI [9]:

if female and S_cr_ ≤0.7 mg/dL: $144\times {\frac{creatinine\;(mg/dL)}{0.7}}^{-0.329}\times 0.993^{age(years)}$

if female and S_cr_ >0.7 mg/dL: $144\times {\frac{creatinine\;(mg/dL)}{0.7}}^{-1.209}\times 0.993^{age(years)}$

if male and S_cr_ ≤0.9 mg/dL: $141\times {\frac{creatinine\;(mg/dL)}{0.9}}^{-0.411}\times 0.993^{age(years)}$

if male and S_cr_ >0.9 mg/dL: 141 × *creatinine* (*mg* / *dL*)^−1.209^ × 0.993^*age*(*years*)^

e) BIS-1 [10]:

$$3.736\times creatinine(mg/dL)\times {age(years)}^{-0.95}\times 0.82\;if\;female$$

### Statistical Analysis

Characteristics of subjects are presented as mean ± SD for continuous variables and percentage of the total for nominal variables. In order to compare the five equations examined using the same ranges, GFR estimates were classified into four categories: ≥60 mL/min/1.73m^2^ (reference category), 45–59 mL/min/1.73m^2^, 30–44 mL/min/1.73m^2^ and <30 mL/min/1.73m^2^. Prevalence of reduced eGFR was defined as the proportion of subjects with an eGFR<60 mL/min/1.73m^2^. The follow-up period in which death from any cause could be ascertained (data from the Registry Office and the Local Health Authority) ranged from 1 day to 5 years after blood collection. Subjects were censored to December 31, 2011. Kaplan-Meier curves adjusted for age groups and sex were used in order to study survival according to categories of eGFR. A test for trend stratified according to age groups and sex was used to examine the possible presence of a gradual effect of eGFR on mortality. Cox proportional hazard regression models were used to calculate hazard ratios (HR) and 95% confidence intervals (CI) using the eGFR ≥60 mL/min/1.73m^2^ as the reference group. To control for the possible confounding effect of other variables on mortality, HRs were adjusted for: age groups, sex, smoke (current or former smoker), BMI (≥30, <18.5), diabetes, hypertension, myocardial infarction, heart failure, stroke and cancer. Age was included as a categorical variable, because the distribution of age was right skewed and the use of age groups protected the analyses from a possible problem with the non linear relationship of age with mortality. In any case, no relevant differences between results obtained using age as a continuous or categorical variable were found.

Analyses were performed using JMP Pro 10 (SAS Institute Inc.), Stata IC 12.1 (Stata Inc.). Results were considered significant if P < 0.05.

The study procedures were carried out in accordance with the principles outlined in the Declaration of Helsinki of 1964 and following amendments. The local research Ethics Committee of the Hospital Health Authority of Novara approved the study. Written informed consent was obtained from each participant prior to blood sampling and interview.

## Results

**Fig 1:**
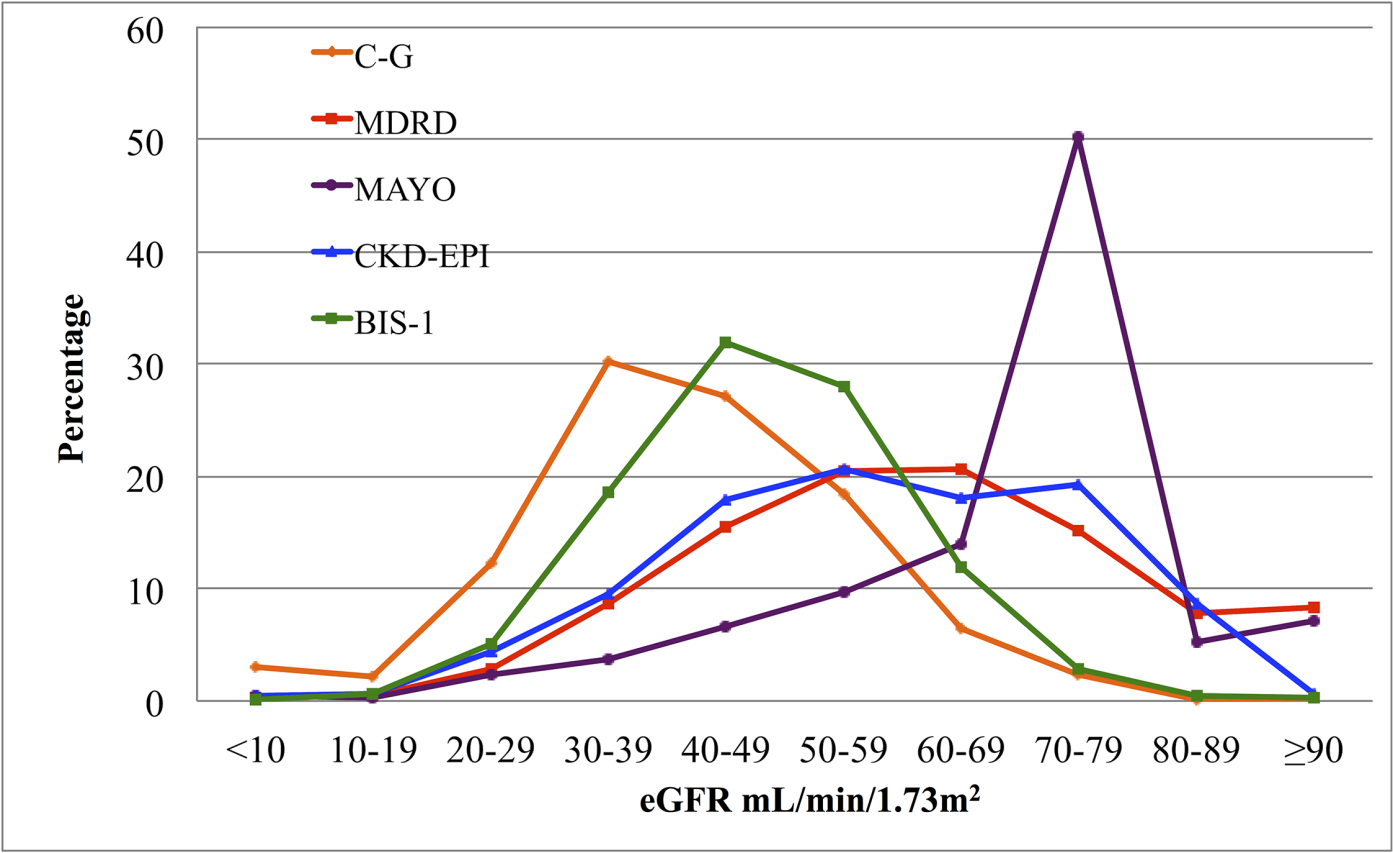
Distribution of estimated Glomerular Filtration Rate (eGFR) according to Cockcroft and Gault equation (C-G); Modification of Diet in Renal Disease (MDRD) formula; MAYO Clinic quadratic equation (MAYO); Chronic Kidney Disease Epidemiology Collaboration (CKD-EPI) formula; Berlin Initiative Study 1 (BIS-1).

**Fig 2:**
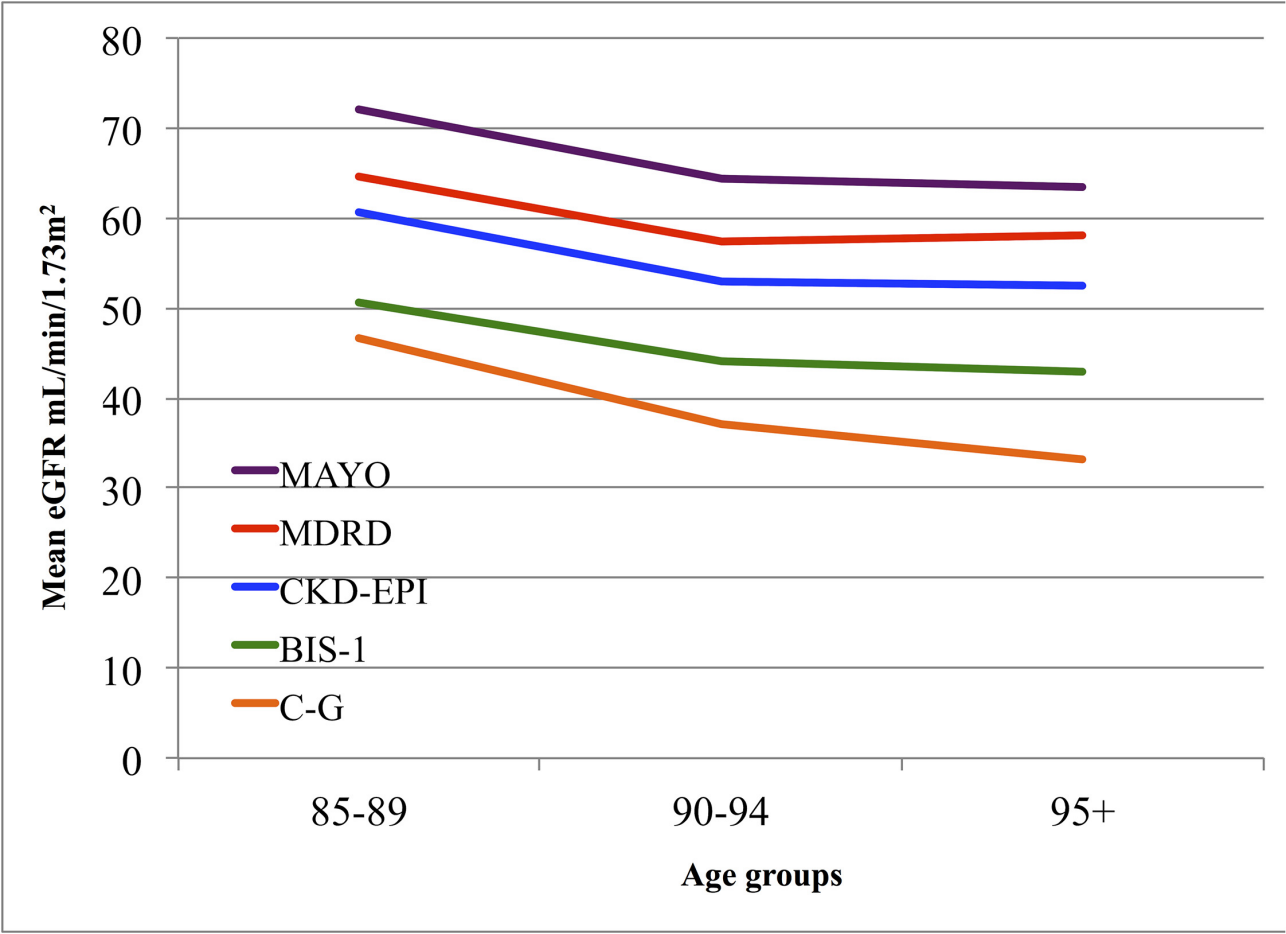
Mean eGFR stratified by age according to Cockcroft and Gault (C-G), Modification of Diet in Renal Disease (MDRD), MAYO Clinic quadratic (MAYO), Chronic Kidney Disease Epidemiology Collaboration (CKD-EPI), and Berlin Initiative Study 1 (BIS-1) equations.

Characteristics of the 700 subjects included in the analyses are set out in Table 1. Mean age of the population was 89.5±3.7 years, with a majority of women (73.3%). During the entire five year period of observation, 316 subjects died (101 men and 215 women), while in the first two years of follow-up 82 subjects died (30 men and 52 women). The frequency distributions of the eGFR according to the five equations in the present study general population show a shift toward higher eGFR using the Mayo equation and toward lower eGFR using both C-G and BIS-1 equations (Fig 1). Mean eGFRs declines with age whatever equation was used to estimate GFR (Fig 2).

**Table 1:** Baseline Characteristics of the Study Population (N between 622 and 700).

***Demographic characteristics and habits***	
Age (years), mean ± SD	89.5 ± 3.7
Sex (Female), n (%)	513 (73.3)
BMI^a^, mean ± SD	23.3 ± 4.3
BMI<18.5, n (%)	81 (12.3)
BMI≥30, n (%)	41 (6.2)
Current smoker, n (%)	25 (3.7)
Former smoker, n (%)	165 (24.4)
***Clinical features***	
Diabetes, n (%)	92 (13.6)
Hypertension, n (%)	418 (62.0)
Angina, n (%)	91 (13.5)
Myocardial infarction, n (%)	56 (8.3)
Heart failure, n (%)	162 (23.2)
Stroke, n (%)	59 (8.7)
Respiratory failure^b^, n (%)	43 (6.9)
Anemia^c^, n (%)	177 (25.3)
Renal failure, n (%)	34 (5.0)
Cancer, n (%)	100 (14.8)
***Deceased after 2-year follow-up*, *n (%)***	82 (11.7)
***Deceased after 5-year follow-up*, *n (%)***	316 (45.1)
***Laboratory analyses***	
Creatinine, mg/dL, mean ± SD	1.0 ± 0.5
BUN^d^, mg/dL, mean ± SD	24.2 ±11.7
Hemoglobin, g/dL, mean ± SD	13.0 ± 1.5
s-Glucose, mg/dL, mean ± SD	109.4 ± 53.4
s-Albumin, g/dL, mean ± SD	4.1 ± 0.4
s-Cholesterol, mg/dL, mean ± SD	196.7 ± 41.5

^a^ BMI, Body Mass Index

^b^ Respiratory failure = use of O_2_ or bronchodilators

^c^Anemia was defined by the WHO criteria as hemoglobin concentration <12.0 g/mL in women and <13.0 g/mL in men

^d^BUN, Blood Urea Nitrogen.

The age-specific prevalence estimates of reduced GFR (<60 mL/min/1.73m^2^) according to the five equations are reported in Table 2. Overall, prevalence estimates of an eGFR <60 mL/min/1.73m^2^ were much higher using the C-G (90.7%) and BIS-1 (84.4%) equations than the CKD-EPI (53.6%), MDRD (48.1%) and Mayo Clinic (23.3%) equations. Independently of the equation used, the prevalence of reduced GFR increased with age, especially between 85 and 94 years (Table 2).

**Table 2:** Prevalence of estimated Glomerular Filtration Rate (eGFR) lower than 60 mL/min /1.73m^2^ according to age.

	C-G	MDRD	MAYO	CKD-EPI	BIS-1
Age Groups (year)	Prevalence (95% CI)				
**85–89** (n = 452; 31.6% men)	86.8 (83.6–90.0)	42.0 (37.4–46.6)	18.6 (15.0–22.2)	46.2 (41.6–50.8)	80.3 (76.6–84.0)
**90–94** (n = 180; 21.7% men)	97.7 (95.5–99.9)	60.0 (52.8–67.2)	31.7 (24.9–38.5	67.8 (61.0–74.6)	91.1 (86.9–95.3)
**≥95** (n = 68; 7.4% men)	98.4 (95.3–101.5)	57.4 (45.6–69.2)	32.4 (21.3–43.5)	64.7 (53.3–76.1)	94.1 (88.5–99.7)
**Overall eGFR <60 mL/min/1.73**	90.7 (88.4–92.9)	48.1 (44.4–51.8)	23.3 (20.2–26.4)	53.6 (49.9–57.3)	84.4 (81.7–87.1)

C-G = Cockcroft and Gault equation; MDRD = Modification of Diet in Renal Disease formula; MAYO = MAYO Clinic quadratic equation; CKD-EPI = Chronic Kidney Disease Epidemiology Collaboration formula; BIS-1 = Berlin Initiative Study 1; CI: Confidence Intervals

Whatever the equation used, the 526 subjects with a history of hypertension, myocardial infarction, diabetes, heart failure, or stroke showed a mean eGFR lower than that of the 151 subjects without, with an average difference between groups of 5.8 mL/min/1.73m^2^ (p = 0.003 using C-G and p <0.0001 using all the other equations) (see Table 3).

**Table 3:** Mean (SD) estimated glomerular filtration rate (eGFR) of oldest old with and without vascular diseases/risk factors ^1^.

Without vascular diseases/Risk factors^1^ n = 151		With at least one disease/Risk factor^1^ n = 526	
	eGFR (mL/min/1.73m^2^)	eGFR (mL/min/1.73m^2^)	P
**C-G** ^**2**^	45.7 (14.9)	42.1 (12.5)	0.003
**MDRD**	68.8 (21.0)	60.5 (18.9)	<0.001
**MAYO**	74.1 (13.6)	68.1 (16.3)	<0.001
**CKD-EPI**	63.0 (15.4)	56.6 (16.4)	<0.001
**BIS-1**	52.0 (12.3)	47.3 (11.5)	<0.001

C-G: Cockcroft and Gault equation; MDRD: Modification of Diet in Renal Disease formula; MAYO: MAYO Clinic quadratic equation; CKD-EPI: Chronic Kidney Disease Epidemiology Collaboration formula; BIS-1: Berlin Initiative Study 1; CI: Confidence Intervals

^**1**^ Vascular diseases/risk factors: diabetes, hypertension, heart failure, myocardial infarction, and stroke.

^2^ n = 144 (without vascular diseases/risk factors) and n = 515 (with vascular diseases/risk factors).

When considering only creatinine as a predictor of mortality, in the present study population subjects with creatinine levels above 1.2 mg/dL were at higher risk of mortality than those with creatinine levels ≤1.2 mg/dL (adjusted HR: 1.69, 95% CI: 1.27–2.23).

**Fig 3:**
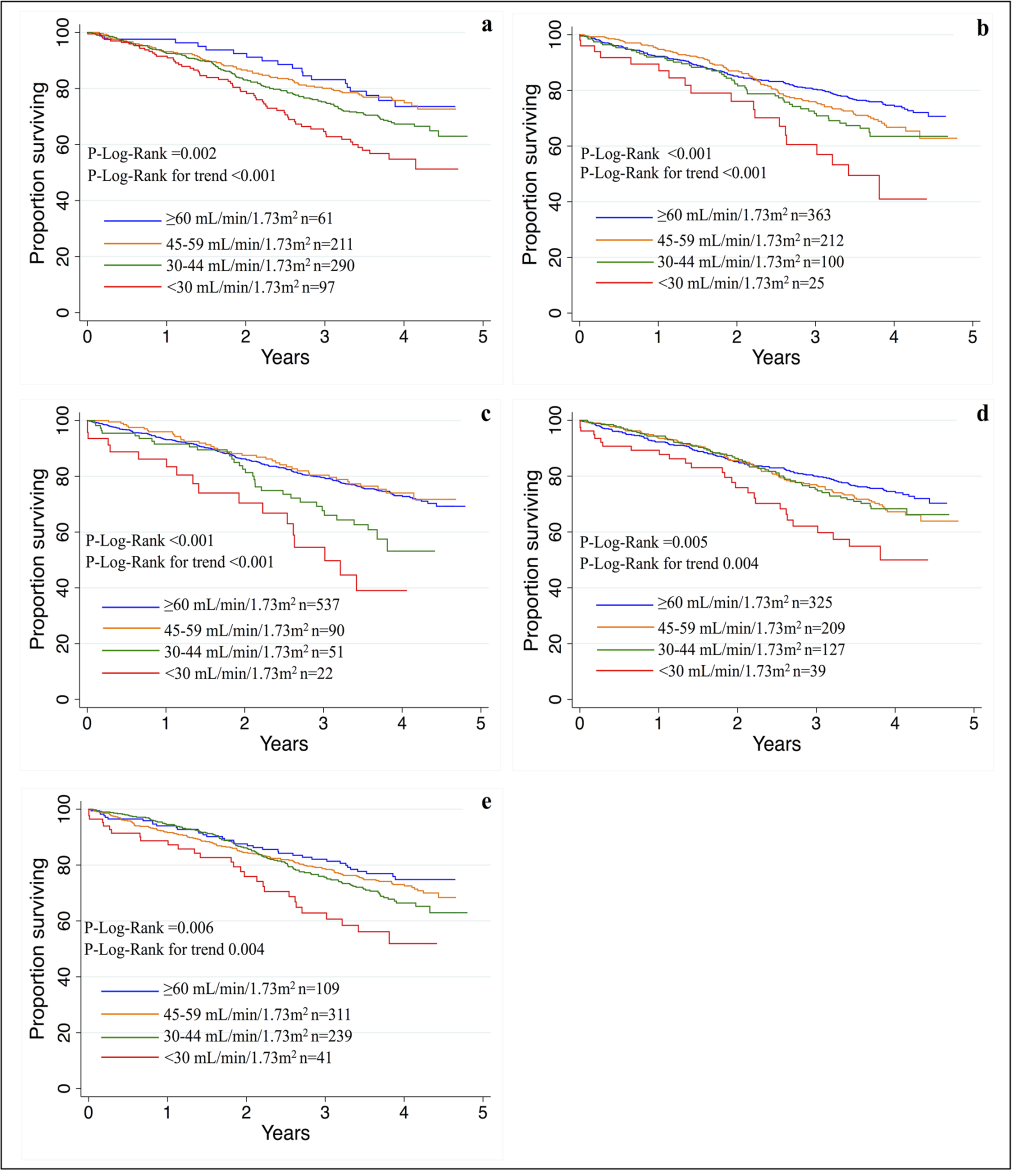
Age groups and sex adjusted survival by estimated Glomerular Filtration Rate (eGFR) according to a) Cockcroft and Gault equation (C-G); b) Modification of Diet in Renal Disease (MDRD) formula; (c) MAYO Clinic quadratic equation (MAYO); d) Chronic Kidney Disease Epidemiology Collaboration (CKD-EPI) formula; e) Berlin Initiative Study 1 (BIS-1).

Age and sex adjusted Kaplan-Meier curves for survival of subjects with an eGFR between 45 and 59, 30 and 44 and <30 mL/min/1.73m^2^ compared to subjects with an eGFR ≥60 mL/min/1.73m^2^ are shown in Fig 3: over the five years of follow-up, eGFR showed a gradual effect on mortality whatever equation was used (age and sex-adjusted P-Log-rank for trend between 0.004 and <0.001). Subjects with severely reduced eGFR (<30 mL/min/1.73m^2^) were at higher risk of mortality with all the five equations.

Univariate and adjusted HRs for mortality after two and five years of follow-up are reported in Tables 4 and 5. After adjustment for other possible confounders, two-year mortality was significantly associated with reduced GFR estimated only with C-G and BIS-1 equations (HRs: 3.30 and 2.88, respectively). After five years of follow-up, in the adjusted models the effect of reduced eGFR on all-cause mortality was significant when GFR was estimated with MDRD, MAYO, CKD-EPI (adjusted HRs 1.43, 1.55 and 1.32, respectively) and, marginally with BIS-1 (HR: 1.45, p = 0.056). Compared to subjects with eGFR ≥60 mL/min/1.73m^2^, subjects with eGFR <30 mL/min/1.73m^2^ were at higher risk of mortality with any equation used (HRs between 2.04 and 2.70). Subjects with eGFR between 30 and 44 mL/min/1.73m^2^ were at higher risk of mortality compared to subjects eGFR ≥60 mL/min/1.73m^2^ when it was estimated with the MDRD, MAYO and BIS-1 equations, while those with eGFR between 45 and 59 mL/min/1.73m^2^ only when GFR was estimated with the MDRD equation.

**Table 4:** Univariate and adjusted Hazard Ratios (HR 95% CI) for 2-year mortality according to Glomerular Filtration Rate (GFR) levels estimated with five different equations (mL/min /1.73m^2^).

	eGFR	N (%)	Deceased n (%)	HR^1^(95% CI)	P^1^	HR^2^(95% CI)	P^2^
**C-G**	≥ 60	61 (9.3)	2 (3.3)	1.00 [Ref]	**0.029**	1.00 [Ref]	0.184
	45–59	211 (32.0)	20 (9.5)	3.06 (0.89–19.14)		3.24 (0.93–20.44)	
	30–44	290 (44.0)	**31 (10.7)**	**3.64 (1.10–22.47)**		3.12 (0.89–19.78)	
	<30	97 (14.7)	**16 (16.5)**	**5.85 (1.66–37.00)**		4.31 (1.13–28.52)	
	<60	598 (90.7)	67 (11.2)	3.76 (0.92–15.36)	0.065	3.30 (1.00–20.40)	0.050
**MDRD**	≥ 60	363 (51.9)	42 (11.6)	1.00 [Ref]	0.151	1.00 [Ref]	0.378
	45–59	212 (30.3)	19 (9.0)	0.78 (0.44–1.32)		0.89 (0.48–1.60)	
	30–44	100 (14.3)	15 (15.0)	1.34 (0.72–2.36)		1.67 (0.83–3.22)	
	<30	25 (3.6)	6 (24.0)	2.19 (0.84–4.78)		1.37 (0.32–4.09)	
	<60	337 (48.1)	40 (11.9)	1.04 (0.68–1.61)	0.842	1.12 (0.67–1.87)	0.653
**MAYO**	≥ 60	537 (76.7)	59 (11.0)	1.00 [Ref]	0.078	1.00 [Ref]	0.436
	45–59	90 (12.9)	9 (10.0)	0.90 (0.42–1.73)		0.84 (0.38–1.67)	
	30–44	51 (7.3)	7 (13.7)	1.34 (0.56–2.74)		1.59 (0.59–3.57)	
	<30	22 (3.1)	7 (31.8)	3.18 (1.32–6.51)		1.99 (0.58–5.18)	
	<60	163 (23.3)	23 (14.1)	1.32 (0.80–2.11)	0.263	1.15 (0.64–1.98)	0.629
**CKD-EPI**	≥ 60	325 (46.4)	37 (11.4)	1.00 [Ref]	0.204	1.00 [Ref]	0.655
	45–59	209 (29.9)	22 (10.5)	0.94 (0.55–1.58)		1.03 (0.56–1.86)	
	30–44	127 (18.1)	14 (11.0)	0.97 (0.51–1.75)		1.07 (0.52–2.11)	
	<30	39 (5.6)	9 (23.1)	2.26 (1.02–4.47)		1.88 (0.67–4.49)	
	<60	375 (53.6)	45 (12.0)	1.08 (0.70–1.67)	0.742	1.11 (0.67–1.87)	0.684
**BIS-1**	≥ 60	109 (15.6)	7 (6.4)	1.00 [Ref]	**0.041**	1.00 [Ref]	0.076
	45–59	311 (44.4)	**41 (13.2)**	**2.13 (1.02–5.20)**		3.03 (1.19–10.24)	
	30–44	239 (34.1)	25 (10.5)	1.68 (0.77–4.22)		2.47 (0.91–8.63)	
	<30	41 (5.9)	**9 (21.9)**	**3.90 (1.45–10.90)**		4.27 (1.16–17.51)	
	<60	591 (84.4)	**75 (12.7)**	**2.06 (1.02–4.92)**	**0.043**	**2.88 (1.16–9.62)**	**0.020**

^1^ Univariate Cox model P-value

^2^ Cox model adjusted for age groups, sex, smoke (current smoker, former smoker), BMI (≥30,<18.5), diabetes, hypertension, myocardial infarction, heart failure, stroke, cancer; C-G = Cockcroft and Gault equation; MDRD = Modification of Diet in Renal Disease formula; MAYO = MAYO Clinic quadratic equation; CKD-EPI = Chronic Kidney Disease Epidemiology Collaboration formula; BIS-1 = Berlin Initiative Study 1

**Table 5:** Univariate and adjusted Hazard Ratios (HR 95% CI) for 5-year mortality according to Glomerular Filtration Rate (GFR) levels estimated with five different equations (mL/min /1.73m^2^).

	eGFR	N (%)	Deceased n (%)	HR^1^(95% CI)	P^1^	HR^2^(95% CI)	P^2^
**C-G**	≥ 60	61 (9.3)	19 (31.1)	1.00 [Ref]	**<0.001**	1.00 [Ref]	**0.003**
	45–59	211 (32.0)	68 (32.2)	1.08 (0.66–1.84)		1.05 (0.64–1.80)	
	30–44	290 (44.0)	**135 (46.5)**	**1.73 (1.10–2.89)**		1.42 (0.88–2.40)	
	<30	97 (14.7)	**63 (64.9)**	**2.96 (1.81–5.08)**		**2.11 (1.24–3.74)**	
	<60	598 (90.7)	**266 (44.5)**	**1.63 (1.06–2.69)**	**0.026**	1.30 (0.83–2.16)	0.267
**MDRD**	≥ 60	363 (51.9)	142 (39.1)	1.00 [Ref]	**<0.001**	1.00 [Ref]	**0.012**
	45–59	212 (30.3)	**103 (48.6)**	**1.30 (1.01–1.68)**		**1.33 (1.01–1.75)**	
	30–44	100 (14.3)	**52 (52.0)**	**1.55 (1.12–2.12)**		**1.56 (1.08–2.21)**	
	<30	25 (3.6)	**19 (76.0)**	**2.63 (1.58–4.14)**		**2.22 (1.21–3.79)**	
	<60	337 (48.1)	**174 (51.6)**	**1.45 (1.16–1.82)**	**<0.001**	**1.43 (1.11–1.84)**	**0.005**
**MAYO**	≥ 60	537 (76.7)	224 (41.7)	1.00 Ref	**<0.001**	1.00 [Ref]	**0.002**
	45–59	90 (12.9)	40 (44.4)	1.10 (0.78–1.53)		1.00 (0.69–1.42)	
	30–44	51 (7.3)	**34 (66.7)**	**1.96 (1.34–2.77)**		**1.70 (1.11–2.50)**	
	<30	22 (3.1)	**18 (81.8)**	**3.08 (1.83–4.83)**		**2.70 (1.47–4.57)**	
	<60	163 (23.3)	**92 (56.4)**	**1.55 (1.21–1.96)**	**<0.001**	**1.55 (1.21–1.96)**	**0.038**
**CKD-EPI**	≥ 60	325 (46.4)	125 (38.5)	1.00 [Ref]	**<0.001**	1.00 [Ref]	**0.038**
	45–59	209 (29.9)	**102 (48.8)**	**1.35 (1.04–1.76)**		1.27 (0.95–1.68)	
	30–44	127 (18.1)	**62 (48.8)**	**1.38 (1.01–1.87)**		1.26 (0.89–1.76)	
	<30	39 (5.6)	**27 (69.2)**	**2.38 (1.53–3.54)**		**2.04 (1.24–3.25)**	
	<60	375 (53.6)	**191 (50.9)**	**1.45 (1.16–1.82)**	**0.001**	**1.32 (1.02–1.70)**	**0.035**
**BIS-1**	≥ 60	109 (15.6)	37 (33.9)	1.00 Ref	**<0.001**	1.00 [Ref]	**0.013**
	45–59	311 (44.4)	130 (41.8)	1.29 (0.91–1.89)		1.30 (0.87–2.01)	
	30–44	239 (34.1)	**121 (50.6)**	**1.69 (1.18–2.47)**		**1.61 (1.06–2.52)**	
	<30	41 (5.9)	**28 (68.3)**	**2.74 (1.66–4.47)**		**2.46 (1.37–4.36)**	
	<60	591 (84.4)	**279 (47.2)**	**1.53 (1.10–2.19)**	**0.011**	1.45 (0.99–2.19)	0.056

^1^ Univariate Cox model P-value

^2^ Cox model adjusted for age groups, sex, smoke (current smoker, former smoker), BMI (≥30,<18.5), diabetes, hypertension, myocardial infarction, heart failure, stroke, cancer; C-G = Cockcroft and Gault equation; MDRD = Modification of Diet in Renal Disease formula; MAYO = MAYO Clinic quadratic equation; CKD-EPI = Chronic Kidney Disease Epidemiology Collaboration formula; BIS-1 = Berlin Initiative Study 1

Using eGFR between 45 and 59 mL/min/1.73m^2^ as the reference category instead of ≥60 mL/min/1.73m^2^, in the shorter period subjects with an eGFR ≥ 60 mL/min/1.73m^2^ had lower risk of mortality with C-G, CKD-EPI and BIS-1 equations and higher risk of mortality with MDRD and MAYO, but none of these results reached statistical significance. In the longer period, subjects with an eGFR ≥ 60 mL/min/1.73m^2^ had lower risk of mortality with all equations, reaching statistical significance in the multivariable analysis of the MDRD equation results (HR: 0.75, 95% CI: 0.57–0.99).

In the present population of oldest old, subjects with eGFR ≥90 mL/min/1.73m^2^ were few: 2 with the C-G, 58 with the MDRD, 50 with the MAYO, 4 with the CKD-EPI, and 2 with the BIS-1. Thus, the association of the highest eGFR with mortality could be explored only using the MDRD and MAYO equations. After adjusting for confounders, in the 2-year and 5-year mortality analyses subjects with a GFR ≥90 mL/min/1.73m^2^ estimated with both equations were not at higher risk of mortality than those with an eGRF of 60–89 mL/min/1.73m^2^ (HRs between 0.08 and 0.92).

## Discussion

In this population-based study, the prevalence of reduced eGFR (<60 mL/min/1.73m^2^) varied markedly according to the equation used: subjects classified in the <60 mL/min/1.73m^2^ category were the majority using the C-G and BIS-1, about half using the MDRD and CKD-EPI, and a minority using the MAYO. Similar differences were observed in other studies in the general elderly population and in subgroups of oldest old, with the C-G and Mayo equations showing, respectively, the highest and lowest estimates while the MDRD and CKD-EPI equations the intermediate estimates [12,25–28]. Prevalences of reduced GFR estimated with the different equations in the present Italian population were similar to those reported for other populations of oldest old from various other countries [12,17,20,29–33]. Prior studies have reported a marked increase in prevalence of low eGFR with age [18, 29–31,34–37]. In the present study, we show that this decrease in eGFR and consequent increase in prevalence continues to grow also in the oldest old, at least up into the nineties years of age.

In the present study population, lower eGFRs were consistently associated with a history of hypertension, myocardial infarction, diabetes, heart failure, or stroke whatever equation was used to estimate GFR. Similar results were found in Nijmegen Biomedical Study using the MDRD equation to estimate GFR in a Dutch population of oldest old (≥85 years) [38].

Several studies have evidenced that reduced GFR is associated with an increased all-cause mortality risk [18, 28–30,39–47]. On the other hand, relative mortality risk associated with each category of GFR seems to decrease with increasing age [29,30,39,40].

The association between reduced GFR and increased mortality has been consistently reported in middle aged and elderly adults [18,30,42,45,46], but only a few studies have investigated this association in the oldest old [17,18, 19, 20, 29, 30]. To our knowledge only one population-based study has attempted to compare the ability of different eGFR equations to predict mortality in oldest old populations. In a cohort of 539 individuals aged 80 years and older (BELFRAIL study), MDRD and CKD-EPI creatinine-based equations showed limited differences in predicting the adverse outcome (death or renal replacement therapy) after an average follow-up period of 2.9 years [17].

In the present population-based study we compared the ability of five creatinine-based equations commonly used to estimate GFR to predict mortality over a shorter and a longer follow-up period. After two years, oldest old with an eGFR<60 mL/min/1.73m^2^ showed an almost three times greater risk of death when GFR was estimated with BIS-1 and C-G equations. However, this increased risk of death was more likely related to the low mortality of the reference group hyper-selected by C-G and BIS-1 equations (3 to 6% versus about 11% using MDRD, MAYO and CKD-EPI) than to the high mortality of the reduced eGFR group (on average 12.0%, a percentage very similar to the 12.7% found using MDRD, MAYO and CKD-EPI). After five years, a sufficient length of time having elapsed, the overall number of deaths (45% of the entire population) made it easier to detect a limited but significant effect of reduced eGFR on mortality. The risk of mortality in fact increased by 30 to 55% in subjects with reduced GFR estimated with all equations, though it reached statistical significance with MDRD, MAYO and CKD-EPI equations (p = 0.056 with BIS1). In this longer period, the rate of mortality was more balanced in the corresponding categories generated by these two groups of equations (31–34% with C-G e BIS-1 versus 39–42% with MDRD, MAYO and CKD-EPI in the reference category, and 45–47% with C-G e BIS-1 versus 51–56% with MDRD, MAYO and CKD-EPI in the reduced GFR category) and the differences in mortality rates between these two categories were thus similar comparing the two groups of equations (on average, 13.5% with C-G and BIS-1 and 13.2% with MDRD, MAYO and CKD-EPI). After five years, increased risk of mortality was significantly associated with severely reduced eGFR regardless of the equation used and with moderately to severely reduced GFR (30–44 ml/min/1.73m^2^) estimated with MDRD, MAYO and, BIS-1 equations. Though mildly to moderately reduced GFR (45–59 mL/min/1.73m^2^) was associated with increased risk of mortality with almost all formulas used to estimate it, only when estimated with the MDRD equation did this association reach statistical significance.

The choice of a “fixed” level of eGFR to identify subjects with possible chronic kidney disease is widely debated, especially in the elderly, in whom GFR declines with age without necessarily being a sign of disease [48–51] However, using a lower eGFR (between 45 and 59 mL/min/1.73m^2^) instead of ≥60 mL/min/1.73m^2^ as a reference category, oldest old in eGFR ≥ 60 mL/min/1.73m^2^ tended to show a lower, though mostly non significant, mortality risk than those in the 45 and 59 mL/min/1.73m^2^, both in the shorter (using C-G, CKD-EPI and BIS-1) and longer period (with all five equations). Thus, in the present population these findings seem to suggest that in the oldest old an eGFR ≥ 60 mL/min/1.73m might be a more effective reference category than 45–59 mL/min/1.73m^2^ to predict mortality. In a meta-analysis of general population and high risk cohorts, a U shaped association between eGFR and mortality risk was observed in the older age group (≥ 65 years) with a significantly higher risk for eGFR >115 mL/min/1.73m^2^ [47]. In the present study, oldest old with a GFR ≥90 mL/min/1.73m^2^ estimated with both MDRD and MAYO (the only equations classifing enough subjects in the eGFR ≥90 mL/min/1.73m^2^ category) were not at a higher risk of mortality either after two or after five years of follow-up. However, the number of subjects with an eGFR ≥ 100 mL/min/1.73m^2^ was too few (MAYO and CKD-EPI: 0; C-G and BIS-1: 1; MDRD: 25) to reliably investigate a U shaped association between eGFR and mortality risk at the highest eGFRs. It has been suggested that the increased mortality risk observed in some studies at higher eGFR may be attributable to low muscle mass leading to decreased creatinine generation [43,47]. In the present study, when the very few subjects with a BMI <18.5 and an eGFR ≥90 mL/min/1.73m^2^ (C-G: n = 0; MDRD: n = 7; MAYO: n = 2; CKD-EPI: n = 1; BIS-1: n = 1) were excluded from the eGFR reference category, unsurprisingly no differences of note with the original analyses could be observed.

The main strength of the present study is the inclusion of a large representative cohort of oldest old from the general population followed prospectively over a long period of time notwithstanding the short life expectancy of this age segment (in 2008 in Biella, between 6.6 and 2.0 years in women and between 4.9 and 1.7 years in men of 85 and 100 years respectively). To control for potential confounding factors, results were adjusted for the main clinical conditions associated with mortality.

A limitation was that we did not measure urinary albumin or protein excretion: measured GFR might have helped in interpreting the different ability of the diverse equations to predict mortality over the short- and long-term. Nevertheless, finding that eGFR retained an independent predictive value despite this limitation, suggests a possible usage of eGFR in routine clinical practice. Another limitation, common to large scale population-based studies, was that the classification of subjects into each eGFR category was based on only one measurement of serum creatinine. However, with respect to the study objective, it is difficult to see how this limitation could affect the comparison of the predictive ability of the five equations used for estimating GFR. A further limitation expected was the low number of subjects in the severely reduced eGFR category traceable in a population-based study. Nonetheless, we were able to show that severe reduction of eGFR was associated with the highest risk of mortality regardless of the formula used.

Recently cystatin C-based equations have been proposed instead of creatinine-based equations to estimate GFR in general population studies of adults and young elderly [52,53]. Cystatin C has the advantage of being less influenced by muscle mass. Although it has been suggested that in the oldest old the percentage of muscle wasting is generally very high, this would not affect the comparison of the predictive ability of the various creatinine-based equations used to estimate GFR.

In the present population of 700 individuals aged 85 or older in any case, subjects with BMI <18.5 were only 81 (11.6%, 71 women and 10 men) and those with a very low BMI (<16) were very few: 21 (3%). Considering that serum creatinine is the most commonly used marker to evaluate renal function in clinical practice, the objective of the present study was to compare the ability of different creatinine-based equations estimating GFR to predict mortality in the oldest old. Of course, we cannot exclude that by using cystatin C-based instead of creatinine-based equations to estimate GFR could yield results different from those reported here.

In the oldest old, different equations give rise to quite different estimates of the prevalence of reduced GFR. In this age segment, on the short term an increased risk of death was associated with reduced eGFR only when GFR was estimated with BIS-1 and, to a lesser extent, C-G equations. On the long term the ability of the five equations to predict mortality was not that different and decreasing eGFR showed an increased effect on mortality whatever equation was used even though only when the oldest old were classified using the MDRD equation was this effect significant also for values of eGFR between 45 and 59 mL/min/1.73m^2^. Remarkably, over the 5-year follow-up a very low eGFR (<30 mL/min/1.73m^2^) was consistently associated with a more than doubled risk of death regardless of the equation used.

If replicated, these findings could suggest a possible complementary use in clinical practice and research of the diverse equations available for estimating GFR to identify oldest old at lower and higher risk of death in the short and long period and to assist the physician in dosing drugs eliminated by the kidney in a population with high prevalence of drug usage and at high risk of adverse drug effects.
